# Domestic Arts and Housing

**Published:** 1920-09-25

**Authors:** 


**THE PUBLIC WELL-BEING—*(continued)*.**

### Domestic Arts and Housing.

It is remarkable how in these days almost every question of public health is related in some way or other to the housing question. We have before us at this moment reports of local authorities, housing bodies, sanitary organisations, lectures by medical officers, and articles by economists, all discussing various problems of public health; and there is not a single instance in which the housing factor is not mentioned as one of great importance.

It is to be observed that Sir James Crichton-Browne, in his presidential address last week to the Sanitary Inspectors' Association at Margate, directed their attention to the decline of the domestic arts. In his opinion, "during the industrial period in England the working-classes have to a large extent, owing to the nature of their occupations, lost touch of those domestic arts in which the comfort, happiness, refinement, and healthfulness of life so much depend, and in these arts they must now be instructed." He suggested that the working women of Burnley had shown more wisdom than the bishops in conference at Lambeth in coming to the conclusion that "they must leave the mills to the men and stay at home to look after the babies." Now this subject of the domestic arts is almost as old as civilisation. It has certainly become a vitally interesting matter at the present time, and Sir James Crichton-Browne did good service in emphasising certain aspects of the problem in his vivacious and suggestive way. But one cannot help thinking that to insist on the necessity of concentrating attention on keeping the home beautiful at a time when hundreds of thousands of people have no home to keep beautiful, and while the rest are living in a state of overcrowding which is demoralising and indecent, is to put the cart before the horse.

In Glasgow there are 13,195 houses certified by the medical officer of health as insanitary and unfit for occupation. Yet practically all these houses are occupied. It is not difficult to understand, as the *Glasgow Evening News* points out, why small-pox and infectious diseases of all kinds are rife in that city. It is also worth recalling that the Committee appointed to consider the principles to be followed in dealing with unhealthy areas has issued an interim report relating to London. It advises as the real remedy for unhealthy overcrowding the removal of the surplus population "to garden cities, consisting of dwellings, for less than fifty thousand inhabitants. But these cannot obviously "materialise" for a long time, and the difficulty would be to make it convenient for the surplus population of workers to live so far away from their work. It is significant that, while in the case of six London County Council schemes which displaced tenants during rebuilding, only one in fifty of the old inhabitants returned to the new houses. Liverpool has made entirely successful efforts to retain its old tenants in new buildings which have replaced unsuitable dwellings.

It is perfectly true, as Sir James Crichton-Browne suggested, that "the slum is often created by the sluggard, and a decent home converted into a hovel by a slovenly and ignorant tenant"; and there is ample reason why the Ministry of Health should be induced to seek more adequate legislative power to enable health officials to deal promptly with tenants who can be proved to be responsible for insanitary conditions in their dwellings. But complaints are being reported in different parts of the country that many new houses put up by local authorities as well as by private builders are so badly built that they are likely to produce serious ill-health among the tenants, and that some have, indeed, become almost uninhabitable. A jerry-built house is about the worst kind of false economy that the nation could indulge in. This may be a truism; but its truth is violated so persistently in practice that the Health Ministry will be kept busy in ensuring that the essential conditions of health are secured for every one of the countless houses that must be built in the next decade if Great Britain is to escape internal disaster.

